# Insilico discovery of novel Phosphodiesterase 4 (PDE4) inhibitors for the treatment of psoriasis: Insights from computer aided drug design approaches

**DOI:** 10.1371/journal.pone.0305934

**Published:** 2024-11-13

**Authors:** Abdullah R. Alanzi, Mohammed S. Alsalhi, Ramzi A. Mothana, Jawaher H. Alqahtani, Moneerah J. Alqahtani

**Affiliations:** 1 Department of Pharmacognosy, College of Pharmacy, King Saud University, Riyadh, Saudi Arabia; 2 Department of Pharmaceutical Chemistry, College of Pharmacy, King Saud University, Riyadh, Saudi Arabia; Cholistan University of Veterinary and Animal Sciences, PAKISTAN

## Abstract

Psoriasis is chronic immune-mediated inflammatory disorder characterized by various comorbidities, erythematous plaques with silvery scale which can lead to psoriatic arthritis. The phosphodiesterase 4 (PDE4) protein is a potential drug target to control Psoriasis. In the current study, pharmacophore-based virtual screening of Diversity library of ChemDiv database was first performed, and then the screened hits were docked to the active site of PDE4 to choose the best binding modes. Forty-six hits generated during the virtual screening were prepared and docked to the PDE4 receptor by SP docking module of glide. The binding affinities of the selected hits were calculated by molecular docking and based on the affinities, ten hits were selected for the bioactivity scores prediction and ADMET analysis. Based on the ADMET profiling, four hits **D356-2630, C700-2058, G842-0420** and **F403-0203** were processed to MD simulations for stability analysis. The outcomes showed that these compounds showed strong binding with proteins with better binding free energies. Based on the results of our study, we proposed that these hits can function as lead in the biological assays and in vitro studies are required to develop the novel drug candidates.

## 1. Introduction

Psoriasis is a prevalent chronic immune-mediated inflammatory disorder characterized by numerous comorbidities, erythematous plaques with silvery scale, and considerable effects on the quality of life [1,2]. People of all ages are impacted by psoriasis, which has a global prevalence of 0.5 to 11.4% in adults and 0 to 1.4% in children [3,4]. Although the precise etiology of psoriasis remains unclear, a complex interplay of immune system, environmental, and genetic factors is thought to be responsible. Tumor necrosis factor (TNF)-α, interleukin (IL)-12, IL-17, IL-22, IL-23, and interferon (IFN)-γ are among the several inflammatory mediators involved in the immunopathogenesis of psoriasis [5,6].

There are several ways to treat psoriasis: topical corticosteroids, calcipotriol, and retinoids; oral conventional systemic treatments that suppress the immune system broadly; and biological therapies that target particular receptors or cytokines to regulate inflammation downstream [6,7]. The severity of the disease often determines the course of treatment; moderate-to-severe diseases are typically controlled with topical therapy, while severe diseases require systemic therapy. Despite being widely used and frequently successful in treating psoriatic plaques, topical corticosteroids (TCS) have long-term side effects that restrict their use, including striae and atrophy [8,9]. The goals of managing psoriasis remain the same regardless of the treatment option selected: to offer a highly effective treatment that targets a range of inflammatory mediators involved in the pathogenesis of psoriasis, while guaranteeing a favorable safety profile and minimal side effects [5].

Among psoriasis treatments, phosphodiesterase-4 (PDE4) inhibitors are emerging as promising options. PDE4 is an enzyme found in cells throughout the body that regulates intracellular signaling by degrading cyclic adenosine monophosphate (cAMP), a critical messenger molecule involved in many cellular processes [5]. Phosphodiesterase 4 (PDE4) inhibitors can effectively treat inflammation in various tissues/organs caused by conditions such as psoriasis, chronic obstructive pulmonary disease (COPD), asthma, and Alzheimer’s disease [10–12]. PDE4 inhibitors used in drug therapy have been shown to have some advantages over conventional formulations, such as increased sensitivity to selective inhibitors, unique tissue distribution, and ease of oral administration. By inhibiting PDE4 activity, cAMP levels rise, leading to suppression of immune cell activation and downregulation of pro-inflammatory cytokines, both of which are critical processes in the pathogenesis of psoriasis [4]. Many PDE4 inhibitors have been discovered over time, and some of these substances have moved on to clinical trials. Adverse events such as vomiting, headache, nausea, weight loss, and depression have been reported, which may limit use in some patients. Because PDE-4 inhibitors are small molecules, they can be applied topically; thus, topical PDE-4 inhibitors are currently being studied with the goal of avoiding systemic side effects [5,13,14].

The discovery of novel PDE4 inhibitors for psoriasis treatment using *insilico* methods requires a combination of computational techniques and molecular modeling. Researchers can efficiently screen large chemical libraries for potential PDE4 inhibitors using advanced computational tools like molecular docking, virtual screening, and molecular dynamics simulations. Virtual screening effectively reduces the enormous number of options to a manageable set of promising compounds by using computational techniques to predict how various molecules will interact with a target protein. This speeds up the early stages of drug development, reduces the need for extensive laboratory experiments, and aids in the identification of novel compounds that would otherwise go undetected using traditional methods. Hence, virtual screening improves the efficiency, speed, and innovation in the drug discovery process [15–17]. Hence, in the current study, virtual screening in combination with molecular docking was used to find possible PDE4 inhibitors.

## 2. Methodology

### 2.1. Development of pharmacophore hypothesis

The crystal structure coordinates of the PDE4 (PDB ID: 7W4X) were obtained from Protein Data Bank (https://www.rcsb.org/). The refined structure of PDE4 protein was subjected to Maestro workspace. The structure was minimized and then the receptor cavity based pharmacophore model was developed by Phase tool of Schrödinger [18]. The hypothesis was developed by choosing the receptor cavity and the binding pocket residues were selected manually. Prior to developing the hypothesis, the receptor was prepared by following the steps explained in section 2.4.

### 2.2.Compound library preparation and virtual screening

The Diversity library of ChemDiv database containing 38646 compounds was retrieved and processed for database preparation through Phase [18]. To increase the chemical space search, twenty conformers of each compound were generated at pH by Epik [19]. Additionally, the high energy tautomer was removed. After preparing the database, the developed pharmacophore hypothesis was employed for virtual screening. The output of the screening was analyzed by phase screen score. The phase screen score is a combination of RMSD site matching, volume score, and vector alignments. The hits that met with the selection criteria of 1.5 phase score were selected for the molecular docking studies.

### 2.3. Molecular docking studies

The refined structure of PDE4 protein was processed for the preparation for molecular docking preparation wizard [20]. The preparation involved three steps: preprocessing, optimization, and minimization. In the preprocessing, the hydrogen atoms were added, extra chains were removed, the charges were added, and the side chain atoms of residues were fixed. Further, the tautomeric states were generated by PROPKA [21]. The hydrogen atoms were optimized at pH 7.0. and then the energy of structure was minimized by using OPLS2005 forcefield [22]. The processed structure was subjected for grid generation by selecting the predicted binding site residues. The X, Y, and Z coordinates of generated grid were 24.03, 2.80, and -25.75, respectively. Further, the screened hits were prepared by LigPrep tool [23] and docked to the prepared structure.

### 2.4. Prediction of bioactivity scores

The biological activity of the selected compounds was predicted by using the Molinspiration tool (https://www.molinspiration.com/). The bioactivity of a drug can be impacted by the chemical structure of compound, its potency and lastly the selection of target. A potent drug candidate is expected to bind with the target enzyme or receptor with stability and it will deliver the therapeutic benefits after binding with the receptors and produce fewer side effects as compared to the compounds with lower potency [24]. The bioactivity scores of the compounds selected at the docking steps were predicted against the GPCR, the kinase proteins, several ion channels, proteases, and the inhibitors of enzymes.

### 2.5. ADMET analysis

The higher rate of drug erosion is often attributed to issues related to toxicity and poor pharmacokinetics [25]. To address these challenges, ADMET properties are predicted to assess the pharmacokinetic properties and toxicity risks associated with potential drug candidates [26]. This predictive approach also helps in evaluating the likelihood of lead compounds becoming viable oral drugs. In this study, we used Maestro’s QikProp tool [27] to predict the ADMET characteristics of the most promising compounds. Molecular weight, hydrogen bond donors and acceptors, QPlogBB, QPPCaco, QPlogKhsa, QPlogPo/w, and QPlogHERG were significant characteristics. Hydrogen bond donors and acceptors are metrics that quantify the amount of atom centers and hydrogen atoms available for participating in interactions involving hydrogen bonds. The logarithm of the octanol and water partition coefficient is predicted by QPlogPo/w, which provides information about the compound’s membrane permeability and hydrophobicity. QPlogHERG assesses the potential of a ligand to block the hERG potassium channel, providing information about the likelihood of cardiac toxicity. QPPCaco is a model for intestinal absorption that determines a compound’s permeability over the monolayer of Caco-2 cells. The substance’s ability to penetrate the blood-brain barrier and reach the central nervous system is indicated by QPlogBB, which forecasts the BBB partition coefficient’s logarithm. Finally, the logarithm of the binding affinity to human serum albumin, a necessary protein that influences drug distribution and binding efficiency, is determined by QPlogKhsa. Similarly, the toxicity profiles of the compounds were predicted by using the ProTox-II webtool (https://tox.charite.de/protox3/). The insilico toxicity prediction of the compounds reduces the cost of experimentation. The toxicity properties such as Hepatotoxicity, Carcinogenicity, Immunogenicity, Mutagenicity, and Cytotoxicity were predicted and compared.

### 2.6. MD simulation

Desmond was employed to conduct Molecular Dynamics simulations lasting 100 ns for selected compounds [28]. The protein and ligand complexes were submitted to Molecular Dynamics simulations to evaluate their stability. MD simulations were used to evaluate the stability of complexes after several stages, including preprocessing, optimization, and reduction. The minimizing process was carried out using the OPLS_2005 force field [22]. The complexes were solvated in a periodic box with a 10 Å size containing the TIP3P water molecules [29]. Neutralization of the systems was done by adding counter ions and the 0.15 M NaCl salt as needed to mimic physiological circumstances. The NPT ensemble was set to a temperature of 300 K and a pressure of 1 atm. Prior to the simulation initiation, the systems underwent a relaxation phase. Trajectories were recorded and saved at 40 ps intervals during the simulation, enabling subsequent analysis of the obtained results.

### 2.7. Binding free energy calculations

The binding free energies of the selected complexes were calculated by employing the Prime-MMGBSA module of Schrödinger [30]. The presence of the counter ions in the system was stripped and the VSGB solvent model along with OPLS_2005 forcefield were employed to calculate the binding free energy (ΔG_bind_). The calculations were conducted by using Eq 1. The binding free energy is the difference between complex free energy and the free energy of protein and ligand. The free energy terms used during the calculation were ΔG_coulomb,_ ΔG_covalent,_ ΔG_Hbond,_ ΔG_Lipo,_ ΔG_Packing,_ ΔG_vdW,_ ΔG_straing_energy,_ and ΔG_Solv_Gb_.


$$\Delta {\text{G}}_{\text{Bind}}=\Delta {\text{G}}_{\text{complex}}-\left(\Delta G_{\text{protein}}+\Delta {\text{G}}_{\text{ligand}}\right)$$ (1)

## 3. Results

### 3.1. Pharmacophore hypothesis development

**Fig 1:**
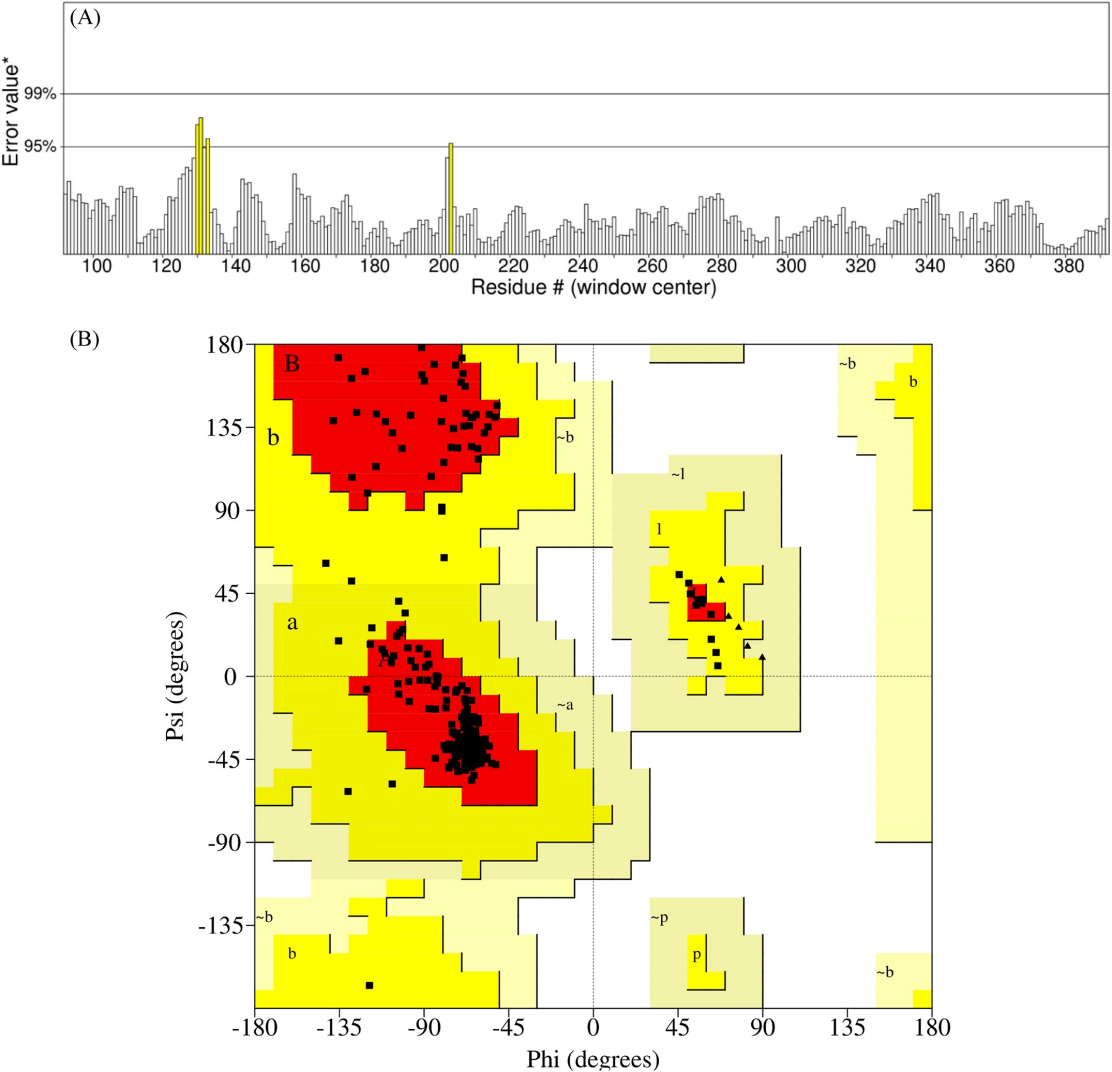
(A) The ERRAT quality factors of PDE4 structure. (B) Ramachandran plot. The yellow region shows the allowed region while the white region shows the disallowed regions.

**Fig 2:**
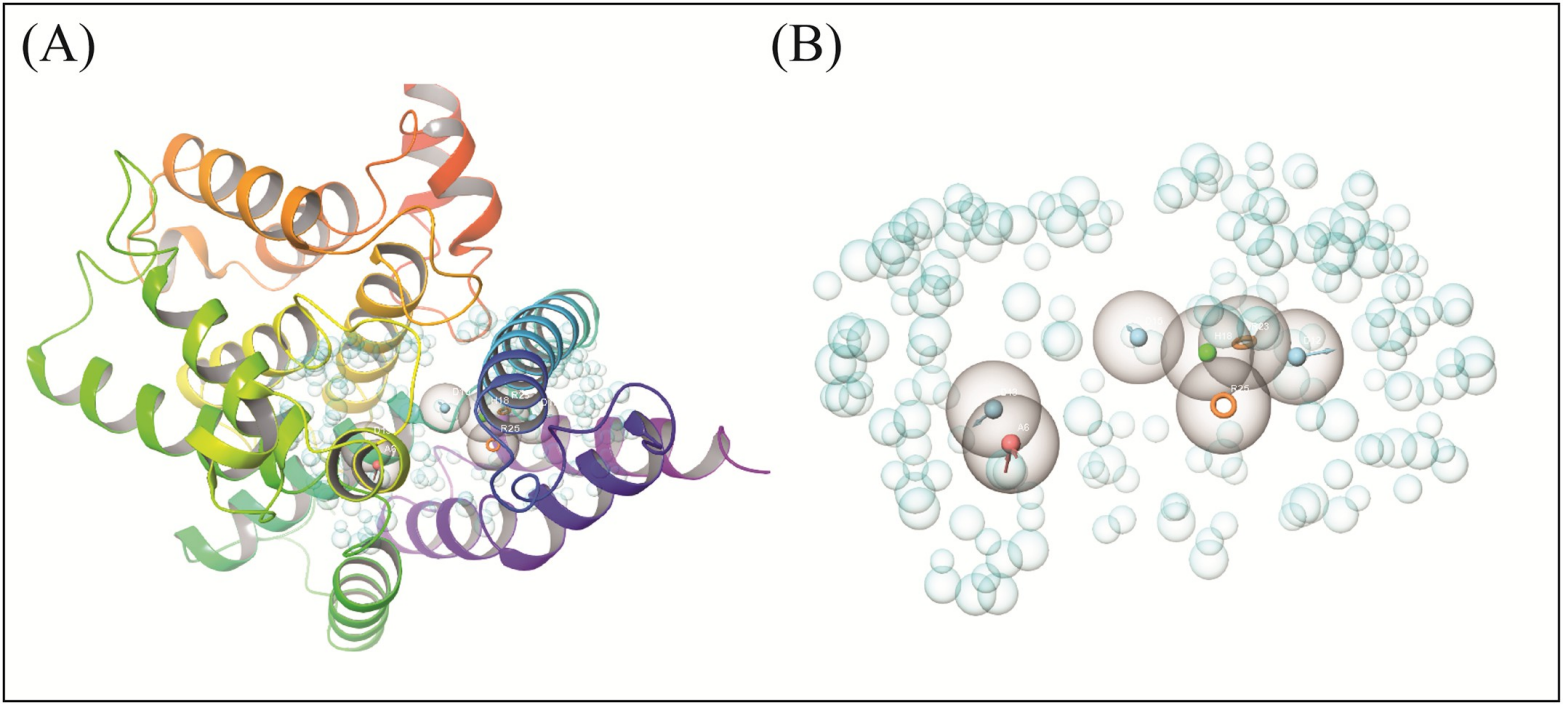
The developed pharmacophore models. (A) The pharmacophoric features shown in the protein pocket. (B) The receptor cavity along with the pharmacophoric features.

The crystal structure was subjected to the workspace of Maestro and optimized for the binding sites prediction. Prior to the binding sites prediction, the structure was validated by calculating the ERRAT quality factor and by observing the Ramachandran plot. A good structure usually shows an average overall quality factor of about 90% for the ERRAT score. The ERRAT quality factor of the PDE4 structure was 98% (Fig 1A). Similarly, the Ramachandran plot of the protein showed that all residues were in the favored region while no residue was observed in disallowed region (Fig 1B). The binding pocket residues were predicted and then selected to generate the pharmacophore hypothesis containing a total of seven features. The pharmacophore hypothesis contained seven features, R1981, A456, H1525, D953, D1041, D963, R2081 (Table 1) shown in Fig 2A and 2B.

**Table 1:** The pharmacophore hypothesis features along with scores and coordinates in the receptor cavity.

Rank	Feature Label	Score	X	Y	Z
1	R1981	-2.81	22.8124	3.0185	-22.3465
2	D953	-2.2	24.9696	4.2669	-21.4469
3	D1041	-2.18	19.6692	0.9717	-25.3118
4	D963	-2.13	21.144	-0.199	-33.1458
5	A456	-1.63	23.4318	0.915	-33.9508
6	H1525	-1.55	23.1533	2.405	-24.241
7	R2081	-1.48	25.1946	4.1546	-25.5467

### 3.2. Virtual screening of CNS library

The developed pharmacophore model was employed for the virtual screening of CNS library of the ChemDiv database. During screening, a compound that matched at least four features was selected as a hit. The screened hits were ranked by the phase screen score which is a combination of Volume score, RMSD site matching, and vector alignments. For better alignment, the vector score must be high in the range of -1.0 to 1.0. Similarly, the reference range for volume is 0.0 to 1.0 where high score shows the greater overlaps among the volume of reference ligand and aligned ligands. It is calculated by dividing the volume of aligned ligands by the volume of the total volume of two ligands, a zero score shows no reference ligand. The potential hits were identified by setting a cutoff phase screen score of 1.5. The compounds with 1.0 phases scores are given in Table 2.

**Table 2:** The hits generated during virtual screening, selected based on the phase screen scores.

No.	ChemDiv IDs	Phase Scores	No.	ChemDiv IDs	Phase Scores
1	7752–0511	1.652	24	8561–08046	1.521
2	7752–0515	1.648	25	D526-0065	1.519
3	8407–0222	1.599	26	D526-0057	1.519
4	D188-0139	1.571	27	D526-0056	1.519
5	8211–0341	1.564	28	D526-0009	1.518
6	8525–0815	1.563	29	D526-0074	1.518
7	8525–0384	1.562	30	D526-0079	1.518
8	8525–0381	1.56	31	D526-0064	1.517
9	D356-2630	1.554	32	D733-0293	1.516
10	8525–0383	1.554	33	D359-0432	1.508
11	D526-0020	1.55	34	D526-0091	1.507
12	D526-0066	1.55	35	F173-0395	1.505
13	G289-0060	1.548	36	D526-0059	1.505
14	D526-0067	1.548	37	D526-0010	1.504
15	D526-0022	1.529	38	D526-0091	1.504
16	G842-0420	1.527	39	F173-0406	1.503
17	D526-0021	1.523	40	F173-0425	1.503
18	D526-0075	1.522	41	F403-0203	1.502
19	F403-0096	1.522	42	D526-0091	1.502
20	D526-0068	1.522	43	D526-0020	1.502
21	D356-2542	1.521	44	C202-3703	1.501
22	D526-0055	1.521	45	D526-0138	1.501
23	D359-0006	1.521	46	C700-2058	1.5

### 3.3. Molecular docking

**Fig 3:**
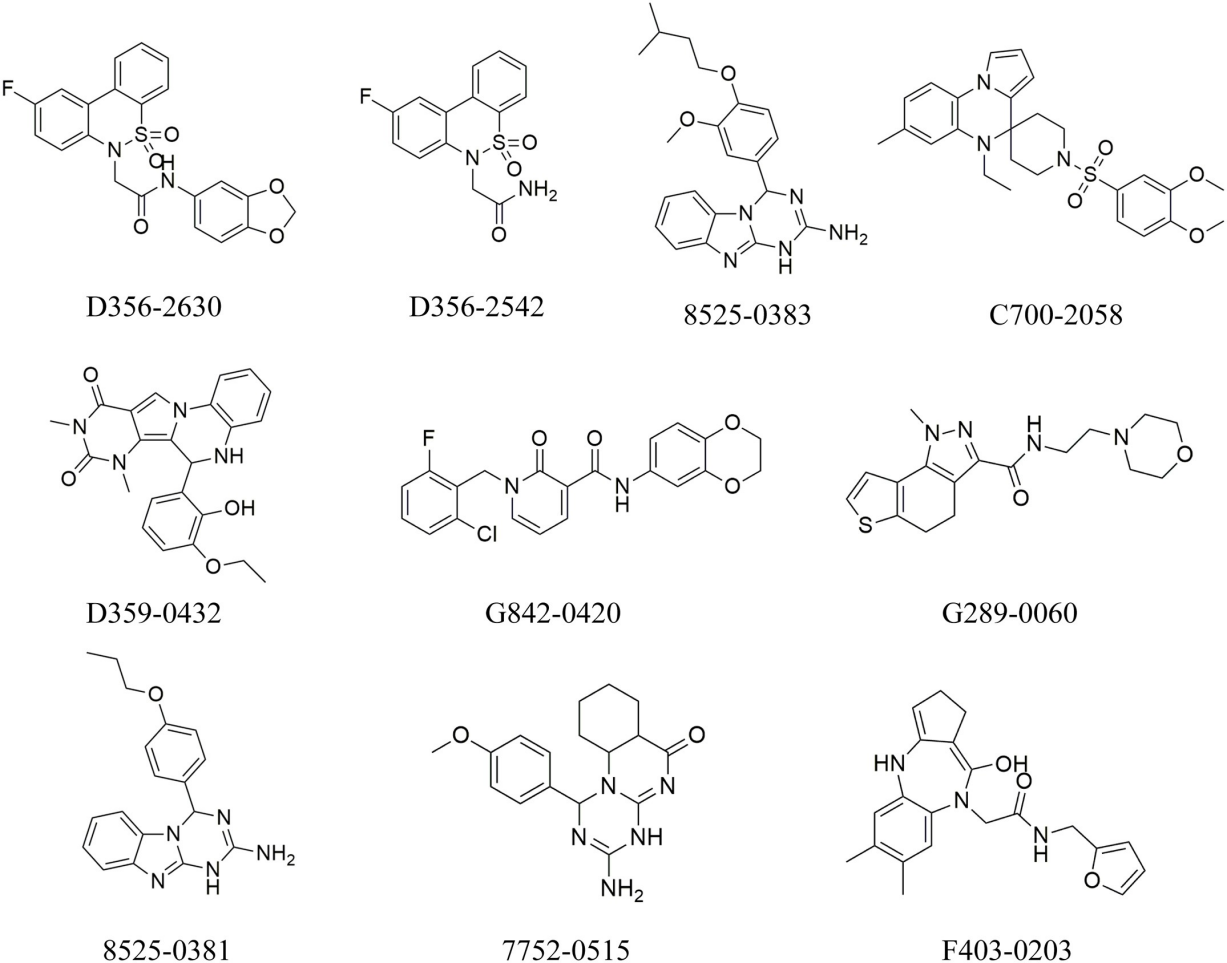
The molecular structures of the compounds selected based on the binding affinities.

**Fig 4:**
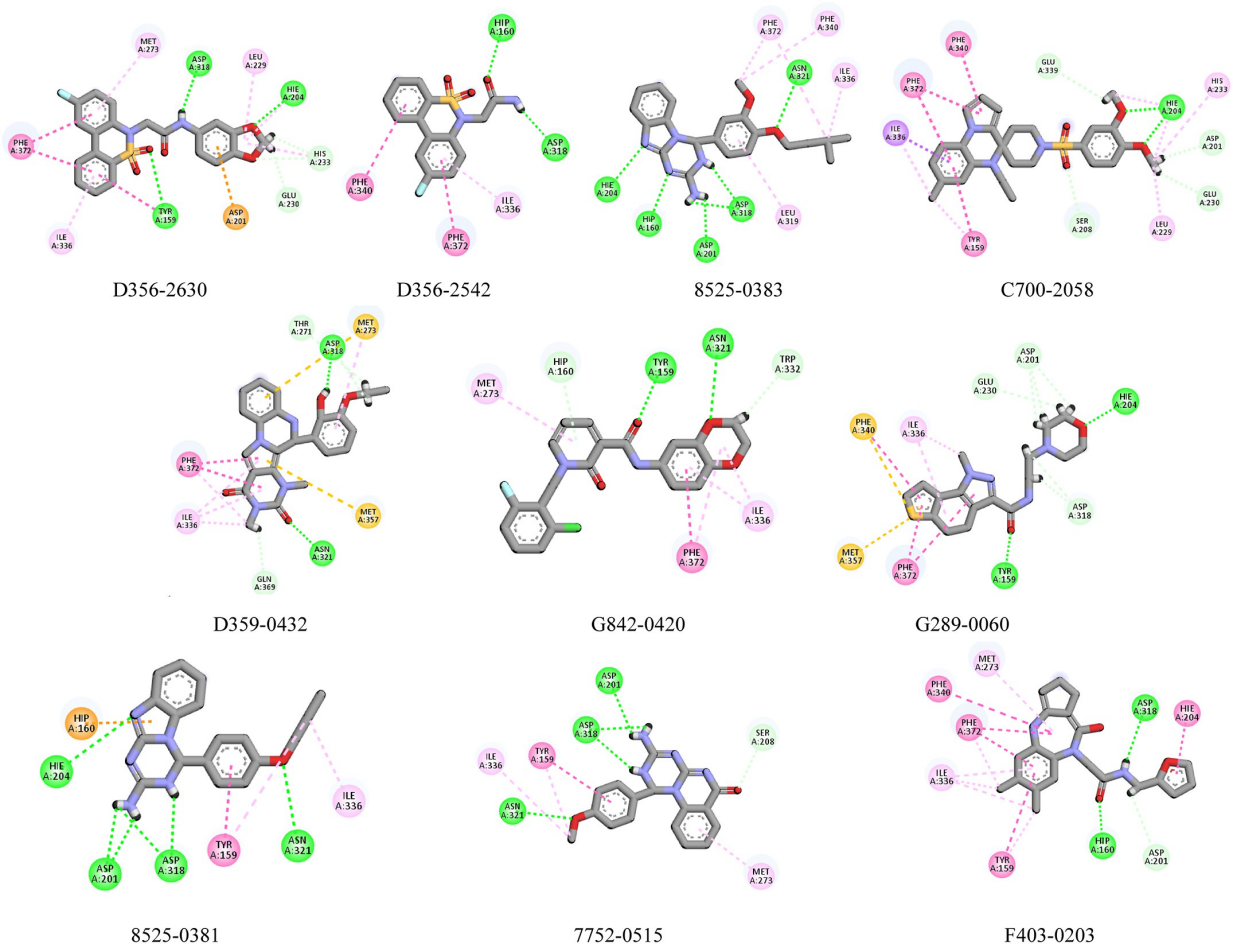
The binding interactions of hits with protein. The hydrogen bonds are shown by green spheres, hydrophobic interactions are shown by magenta color, the purple lines show pi-sigma, while orange lines show the pi-sulfur interactions.

**Fig 5:**
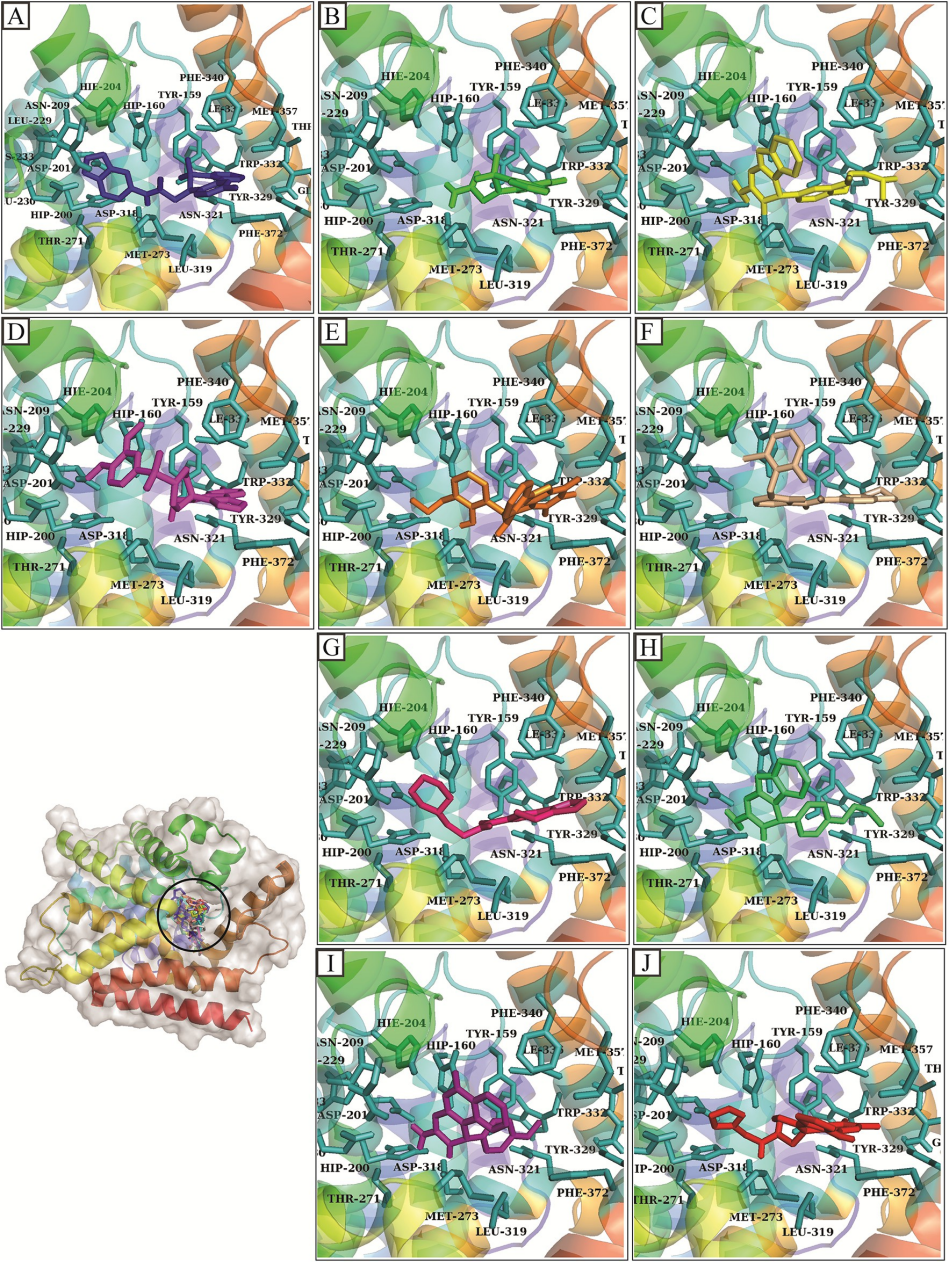
The plausible binding modes of the selected compounds are represented with the sticks in the binding pocket of protein. The orientation of the hits is also shown in the pocket separately. (A) D356-2630 (B) D356-2542 (C) 8525–0383 (D) C700-2058 (E) D359-0432 (F) G842-0420 (G) G289-0060 (H) 8525–0381 (I) 7752–0515 (J) F403-0203.

The hits generated during the virtual screening were prepared and docked to the PDE4 receptor by SP docking module of glide. The docking poses were analyzed based on the glide gscore and molecular interactions of the docked compounds. The compounds with binding affinities greater than -7 kcal/mol were selected for further analysis. The two-dimensional structures of the selected hits are shown in Fig 3. The molecular interactions of the selected docked hits were analyzed, and it was observed that **D356-2630** made five hydrogen bonds with Asp318, His204, Tyr159, His233, Glu230 and five hydrophobic interactions with Phe372, Ile336, met273, Leu229, and Asp201. **D356-2542** made two hydrogen bonds with Asp318, His160 and three alkyl interactions with Phe340, Phe372, and Ile336. **8525–0383** was engaged in hydrogen bonding with Asp318, His204, His160, Asp201, Asn321. It also made hydrophobic interactions with Phe372, Phe340, Ile336, Leu319. Similarly, **C700-2058** made five hydrogen bonds with Glu339, His204, Asp201, Glu230, Ser208. **D359-0432** made hydrogen bonds with Asp318, Gln369, Thr271 during docking while **G842-0420** participated in hydrogen bonding with the residues Asn321, Tyr159, His160, Trp332. **G289-0060** made hydrogen bonds with Asp318, His204, Asp201, Glu230, Tyr159. At last, **8525–0381, 7752–0515, F403-0203** made hydrogen bonds with Asp318, His204, Asp201, Asn321, Asp318, Asp201, Asn321, Ser208, Asp318, His160, and Asp201 (Fig 4). The docking scores, interacting residues, interaction types, and the distance between ligand and hydrogen bond forming residues are given in Table 3. Further, the plausible binding modes of the docked ligands were analyzed, and it was observed that all ten selected ligands occupied the same space in the predicted binding pocket of protein (Fig 5).

**Table 3:** The docking scores, interacting residues and the hydrogen bond distances of the selected compounds with protein.

ChemDiv IDs	Glide scores	Hydrogen Bonds Distance (Å)	Hydrophobic Interactions
D356-2630	-8.062	Asp318(2.02), His204(2.19), Tyr159(2.75), His233(2.35), Glu230(2.29)	Phe372, Ile336, met273, Leu229, Asp201
D356-2542	-7.99	Asp318(1.77), His160(2.03)	Phe340, Phe372, Ile336
8525–0383	-7.942	Asp318(1.65), His204(2.76), His160(2.34), Asp201(1.94), Asn321(2.83)	Phe372, Phe340, Ile336, Leu319
C700-2058	-7.853	Glu339(2.58), His204(1.86), Asp201(2.36), Glu230(2.46), Ser208(4.06)	Ile336, Phe372, Phe340, tyr159, Leu229, His233
D359-0432	-7.779	Asp318(2.06), Gln369(2.31), Thr271(2.72)	Phe372, Ile336, Met357, Met273
G842-0420	-7.317	Asn321(2.61), Tyr159(2.19), His160(2.80), Trp332(2.30)	Phe372, Ile336, Met273
G289-0060	-7.298	Asp318(2.13), His204(1.86), Asp201(2.97), Glu230(279), Tyr159(2.40)	Phe340, Met357, Phe372, Ile336
8525–0381	-7.286	Asp318(2.04), His204(2.61), Asp201(1.94), Asn321(2.70)	His160, Tyr159, Ile336
7752–0515	-7.088	Asp318(1.95), Asp201(2.26), Asn321(2.49), Ser208(3.49)	Ile336, Tyr159, Met273
F403-0203	-7.07	Asp318(1.89), His160(2.12), Asp201(2.90)	Phe340, Met273, Phe372, Ile336, Tyr159, His204

### 3.4. Bioactivity scores

The bioactivity scores of the compounds help to analyze the drug binding ability to various human receptors. The bioactivity scores of the selected seven compounds were predicted by Molinspiration tool. The predicted scores of the compounds against several receptors are shown in Table 4, indicating that the scores were in the range of -5 to 0.0. showing the moderate activity of these compounds against the receptors. Some compounds showed scores close to zero, indicating good activity against the receptors. The analysis revealed that the selected compounds have the properties of lead compounds.

**Table 4:** The predicted bioactivity scores of the selected compounds against different human receptors.

ChemDiv IDs	GPCR ligand	Ion channel modulator	Kinase inhibitor	Nuclear receptor ligand	Protease inhibitor	Enzyme inhibitor
D356-2630	0.01	-0.23	-0.44	-0.25	-0.01	-0.31
D356-2542	0.14	-0.06	-0.53	-0.19	0.16	-0.26
8525–0383	-0.32	-0.59	-0.51	-0.91	-0.50	-0.44
C700-2058	-0.05	-0.28	-0.40	-0.40	-0.20	-0.31
D359-0432	-0.29	-0.51	-0.69	-0.51	-0.63	-0.37
G842-0420	-0.13	-0.48	-0.04	-0.36	-0.36	-0.23
G289-0060	0.14	-0.42	0.44	-0.30	-0.11	-0.08
8525–0381	-0.38	-0.67	-0.58	-1.00	-0.61	-0.48
7752–0515	-0.51	-0.65	-0.62	-1.00	-0.72	-0.46
F403-0203	-0.35	-0.64	-0.74	-0.65	-0.46	-0.36

### 3.5. ADMET analysis

QikProp was used to predict the ADMET characteristics of the selected compounds, and it was found that the expected values fell within an acceptable range. The molecular weight of a compound indicates its easy distribution in the cells so the compounds with less weight can easily distribute in the body as compared to the compounds with higher weight. In this regard, a criterion of 500 g/mol was set, and all the molecular weights of all selected compounds fall within this range. QPlogPo/w determines the octanol/water partition coefficient, a value within a range of 1.0 to 4 is good. The values of selected hits fall within this range. The compounds that were selected had anticipated ADMET qualities that are within the acceptable range, as demonstrated. in Table 5. Similarly, ProTox-II server predicted the toxicity profiles of the compounds. It can be observed that all the hits showed toxicity class 4 except for D356-2542 and C700-2058 which showed toxicity class 6 and 3 respectively (Table 6). Additionally, 8525–0383, G289-0060, 8525–0381, and 7752–0515 showed active profiles for one or two parameters of hepatotoxicity, carcinogenicity, immunotoxicity, mutagenicity, and cytotoxicity. After ADMET analysis, four compounds **D356-2630, C700-2058, G842-0420** and **F403-0203** showed better QPPCaco values so these were selected for further stability analysis.

**Table 5:** The ADMET and toxicity risks analysis of the selected hits.

Compounds	MW	HBD	HBA	QPlogPo/w	QPlogHERG	QPPCaco	QPlogBB	QPlogKhsa
D356-2630	426.418	1	8	2.773	-5.567	1365.029	-0.323	-0.106
D356-2542	306.311	2	7	0.493	-3.4	175.083	-0.837	-0.685
8525–0383	379.461	3	5	3.968	-5.865	653.108	-1.089	0.59
C700-2058	481.609	0	7	4.513	-4.538	1972.66	-0.372	0.481
D359-0432	418.451	2	6	3.836	-6.003	464.089	-1.049	0.675
G842-0420	414.82	0	6	4.197	-6.092	2488.033	-0.107	0.201
G289-0060	346.446	1	7	2.495	-5.419	760.416	0.323	0
8525–0381	321.381	3	4	3.156	-5.75	624.636	-0.918	0.323
7752–0515	321.338	3	6	1.616	-5.114	218.402	-1.137	-0.043
F403-0203	365.431	3	4	3.683	-4.237	1741.637	-0.417	0.291

"QPlogHERG" (<-5), "QPlogPo/w" (-2.0 to 6.5), "QPlogBB" (-3.0 to 1.2), "QPPCaco" (<25 poor, >500 great), and "QPlogKhsa" (-1.5 to 1.5).

**Table 6:** The toxicity profiles of the selected hits.

Compounds	Hepatotoxicity	Carcinogenicity	Immunotoxicity	Mutagenicity	Cytotoxicity	Predicted LD50 (mg/kg)	Toxicity Class
D356-2630	Inactive	Inactive	Inactive	Inactive	Inactive	1250	4
D356-2542	Inactive	Inactive	Inactive	Inactive	Inactive	5800	6
8525–0383	Inactive	Active	Active	Inactive	Inactive	1470	4
C700-2058	Inactive	Inactive	Inactive	Inactive	Inactive	300	3
D359-0432	Inactive	Inactive	Inactive	Inactive	Inactive	1000	4
G842-0420	Inactive	Inactive	Inactive	Inactive	Inactive	540	4
G289-0060	Inactive	Active	Inactive	Inactive	Inactive	1000	4
8525–0381	Inactive	Active	Inactive	Inactive	Inactive	450	4
7752–0515	Active	Active	Inactive	Active	Inactive	1000	4
F403-0203	Inactive	Inactive	Inactive	Inactive	Inactive	1200	4

### 3.6. MD simulation

After the ADMET analysis, four compounds with highest drug-likeness and not toxicity risks were selected for the stability analysis by conducting 100 ns simulation. The MD trajectories were analyzed by measuring the RMSD, RMSF, Protein-ligand contacts, PCA, and drawing the 2D PCA based free energy diagrams.

#### 3.6.1. RMSD

**Fig 6:**
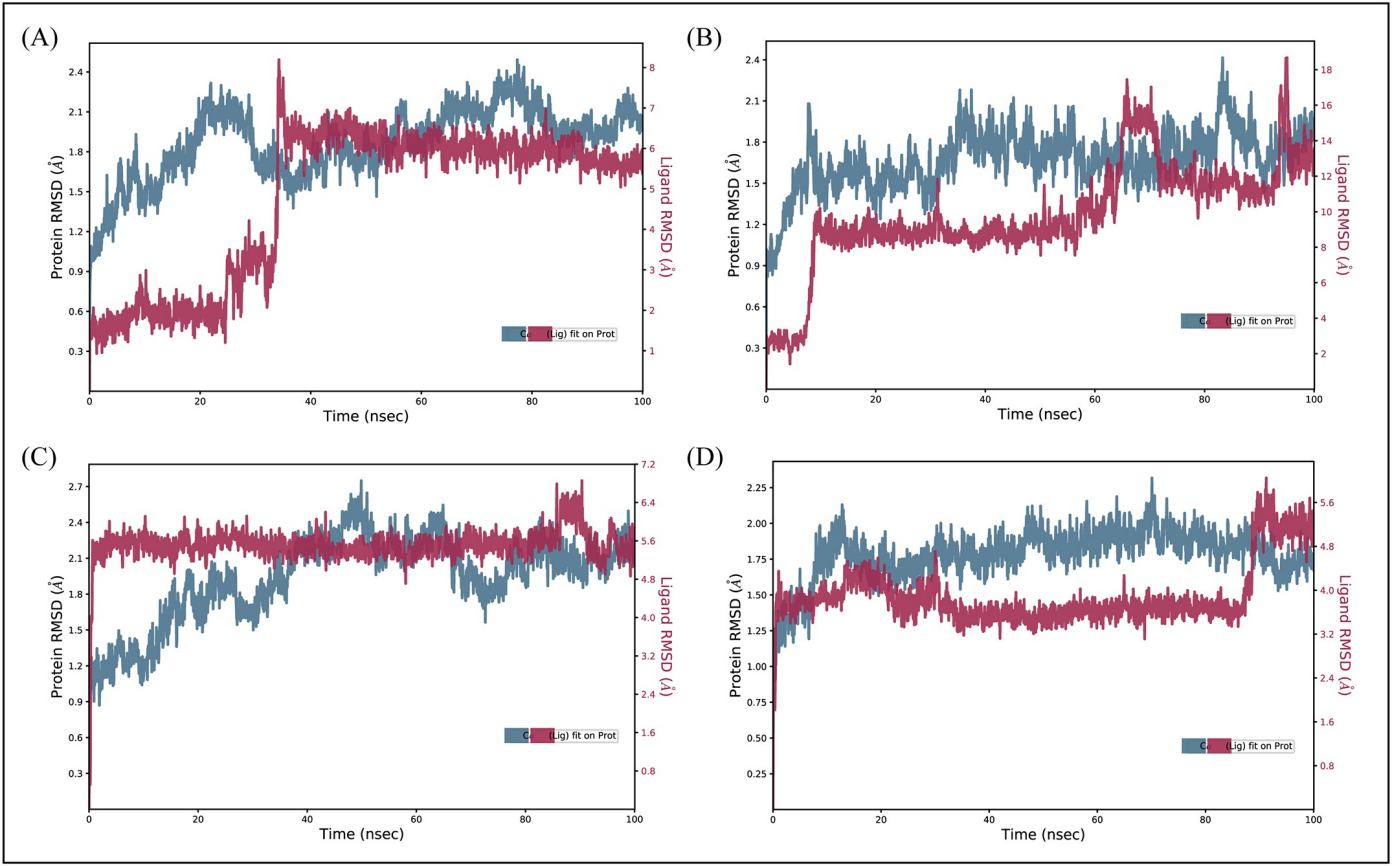
The RMSD protein along with selected hits calculated during simulation. (A) D356-2630 (B) C700-2058 (C) G842-0420 (D) F403-0203.

The RMSD of carbon alpha atoms of protein was calculated and aligned with ligand atoms RMSD to investigate the stability of protein ligand complex [31]. It can be observed that the RMSD of **D356-2630** complex deviated in the range of 0.9–2.1 Å till 30 ns and then attained stability in the range of 1.5–2.4 Å till the end of simulation, while the RMSD of ligand was fitted on the protein (Fig 6A). The RMSD of carbon alpha atoms of **C700-2058** complex started at 0.9 Å and increased to 2.1 Å and then stayed between the range of 1.3–2.4 Å till the end of simulation while the RMSD of ligand atoms remained less than protein (Fig 6B). The RMSD of **G842-0420** (Fig 6C). complex attained stability at 40 ns in the range of two Å with the almost same RMSD value of ligand. Lastly, **F403-0203** (Fig 6D) the RMSD value stabilized in the range of 1.25–2.00 Å with the RMSD of ligand less than of protein. All our findings collectively demonstrate that the protein and ligand complexes stayed stable during MD simulation, providing valuable new insight into their dynamic behavior and suitability for further investigation.

#### 3.6.2. RMSF

**Fig 7:**
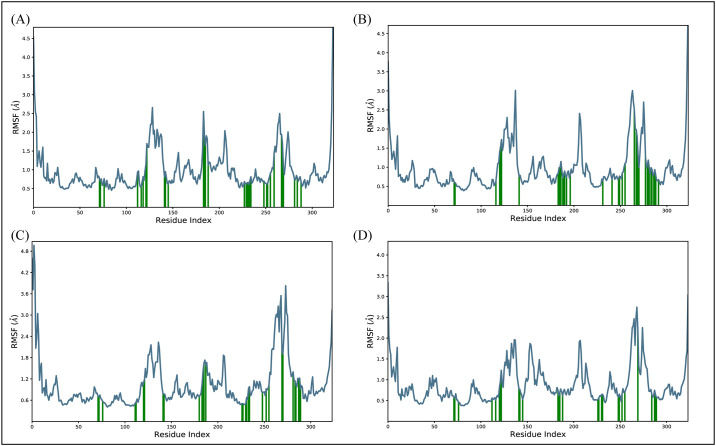
The residual flexibility of the complexes during simulation along with the loop regions with higher fluctuations. (A) D356-2630 (B) C700-2058 (C) G842-0420 (D) F403-0203.

Root mean square fluctuations (RMSF) values have been calculated in order to examine the fluctuating behavior of the proteins while they are bound to the ligands [32]. For each protein residue over the simulation period, RMSF values give detailed information on the residue’s flexibility and mobility. Based on the expected RMSF values, most protein residues changed very slightly during the simulation, measuring less than 3 Å except for the loop regions L1, L2, and L3. This suggests that these residues maintained their relative stability in the presence of ligands. However, the loop regions of the protein exhibited higher RMSF values, which were around 4.5 Å (Fig 7). The residues that form the contacts with the ligand did not undergo any confirmational changes and remained stably bound with the ligands. Overall, RMSF values are compatible with the idea of a stable protein and ligand.

#### 3.6.3. Protein-ligand contacts

**Fig 8:**
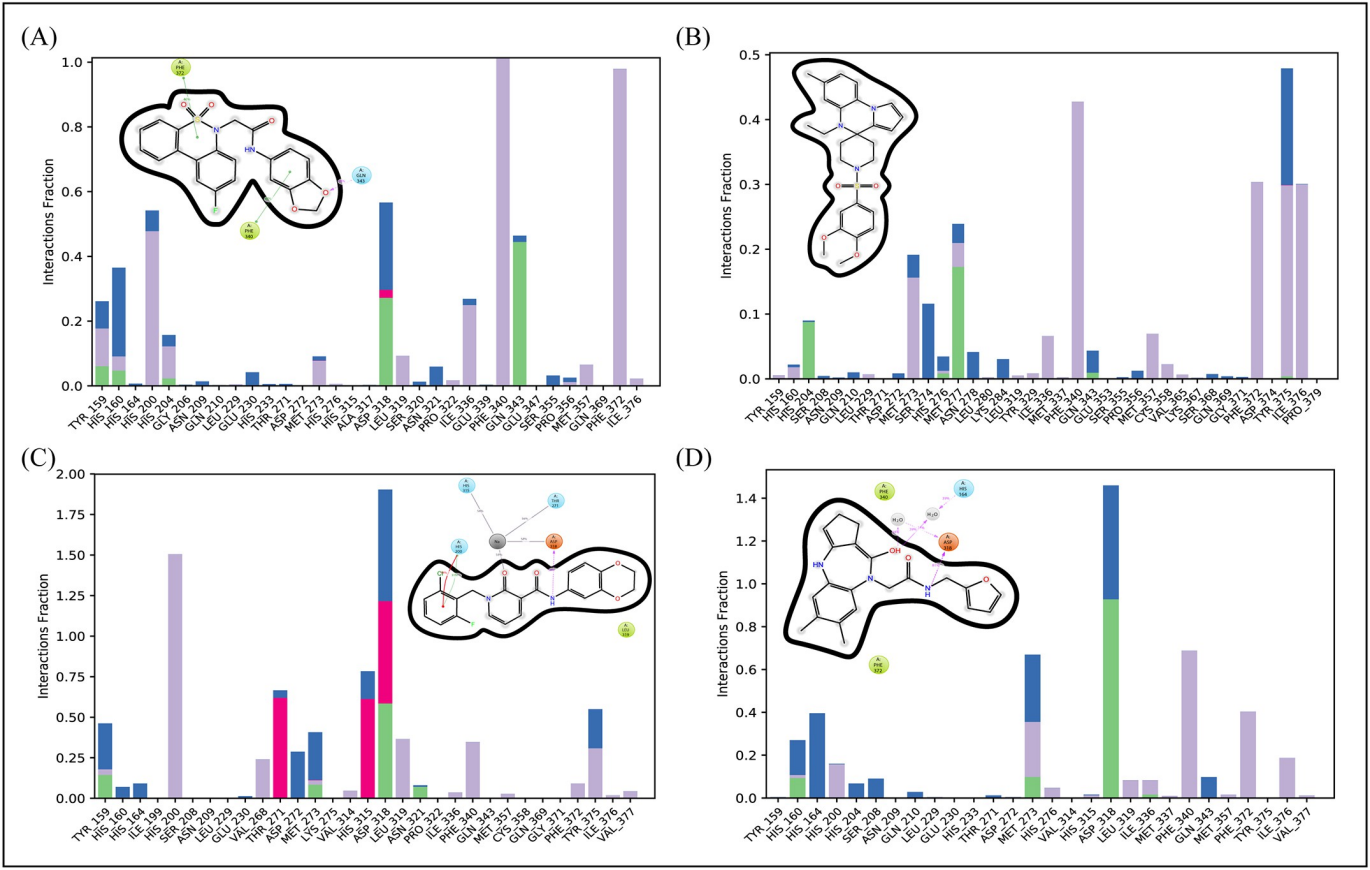
The Protein-Ligand contacts generated during the 100 ns simulation. (A) D356-2630 complex. B) C700-2058 complex. (C) G842-0420 complex. (D) F403-0203 complex. Green shows hydrogen bonding, purple shows hydrophobic interactions, and blue shows the water bridges.

The MD Simulation analysis showed that ionic, hydrogen, and hydrophobic bonds were the most important types of interactions between the ligands and the protein. The functional properties of the protein-ligand complex are stabilized and regulated by these interactions. The residues that form hydrogen bonds with **D356-2630** were Tyr-159, His-160, His-204, Asp-318 and Gln-343.The residue that form ionic bond is Asp-318. (Fig 8A). In the **C700-2058** complex, residues involved in bonding with hydrogen were His-206, His-276 and Met-277 (Fig 8B). The hydrogen bonding in **G842-0420** complex was observed in Tyr-159, Met-273, Asp-318 and Asn-32,1 while Thr-271, His-315, Asp-318 engaged in ionic bond (Fig 8C). Lastly, in **F403-0203** (Fig 8D). residues involved in bonding with hydrogen were Tyr-159, Met-273 and Asp-318. These hydrogen bonding interactions, which were displayed during the MD simulations, not only highlighted the specific residues that were crucial for stabilizing the protein-ligand complexes, but they also provided insight into the crucial interactions that underpin the complexes’ general stability and binding affinity.

#### 3.6.4. Principal component analysis and PCA based free energy

**Fig 9:**
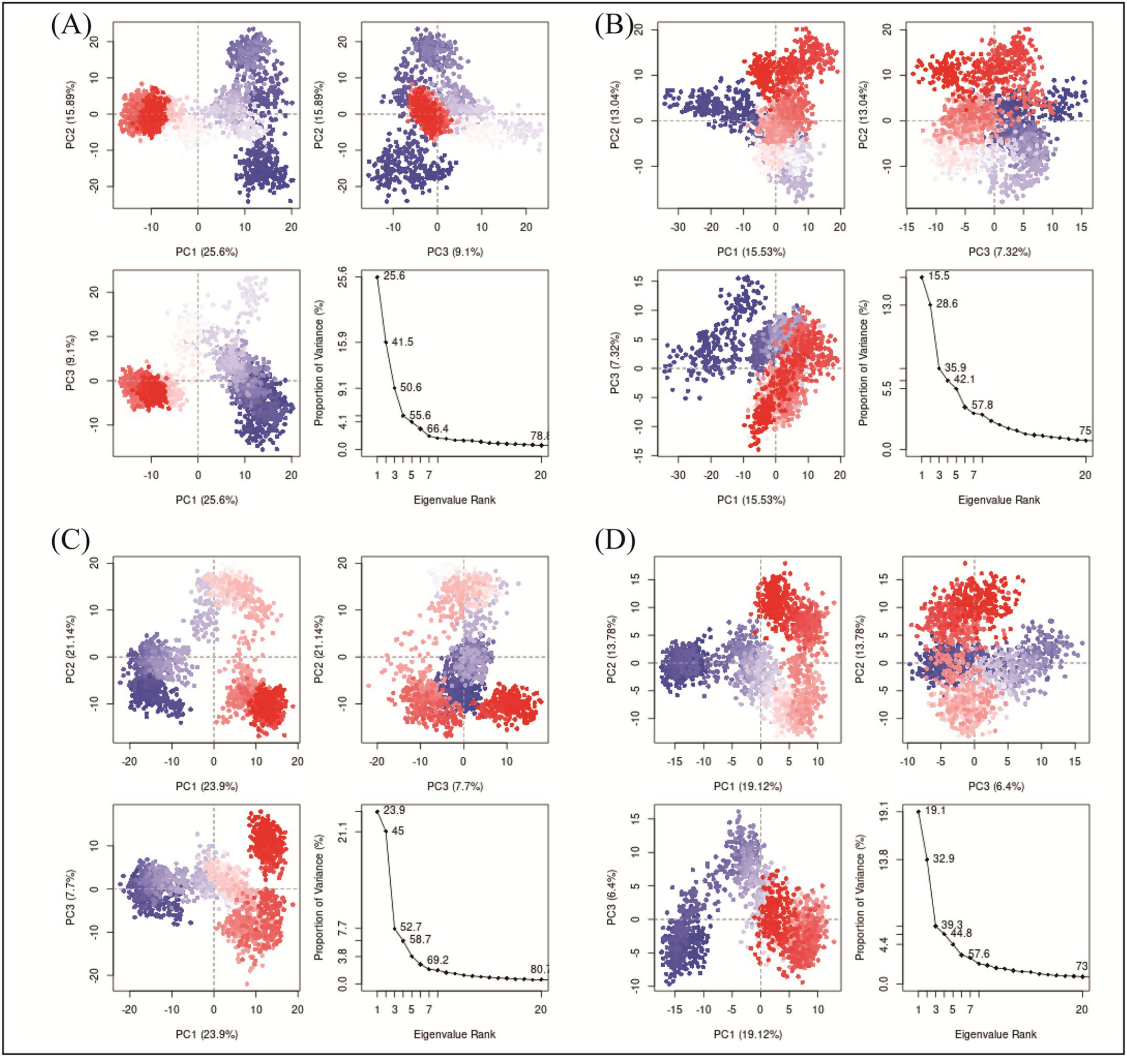
Principal component analysis of the selected complexes. (A) D356-2630 B) C700-2058 (C) G842-0420 (D) F403-0203.

**Fig 10:**
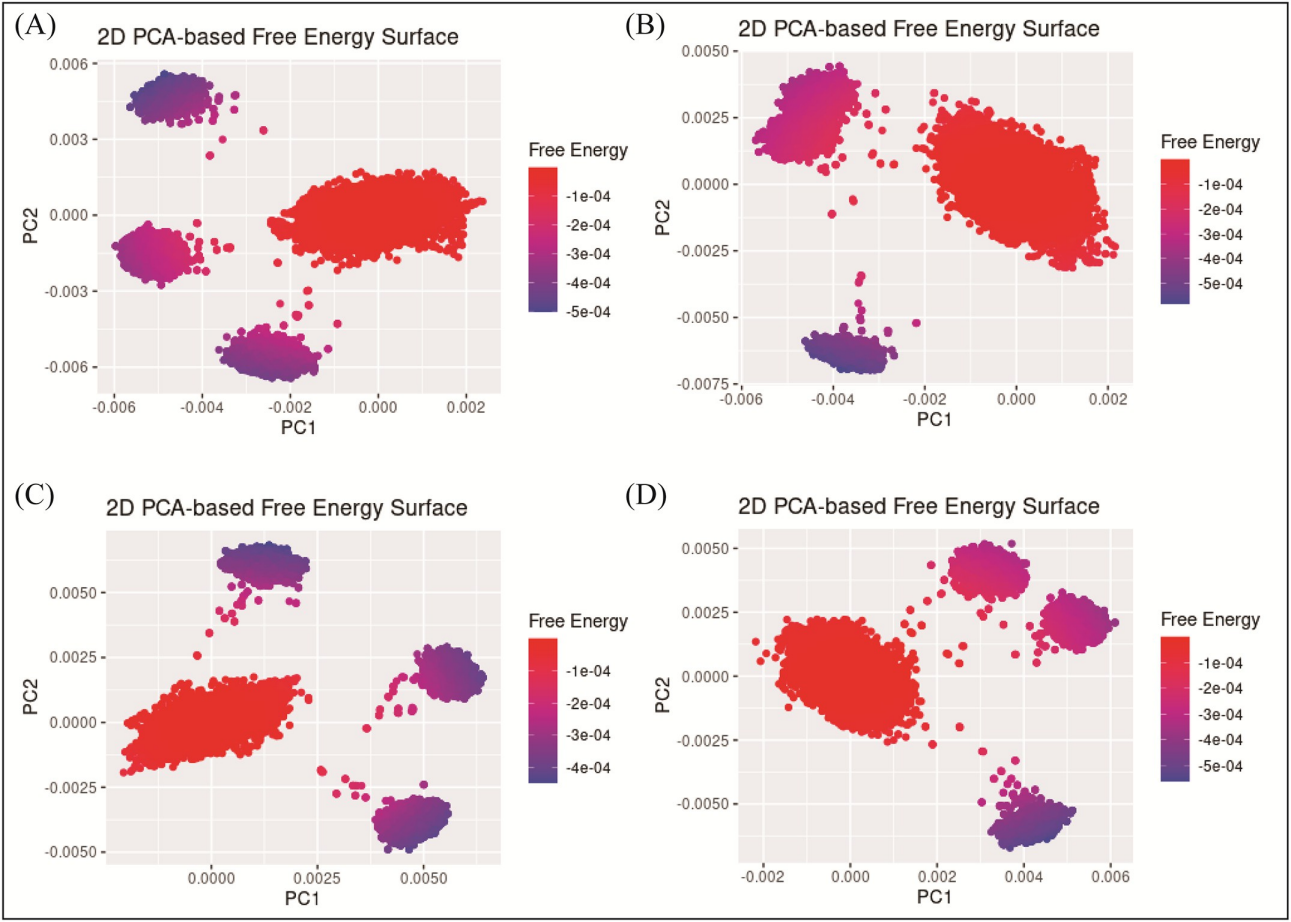
PCA based free energy surface of the complex calculated during simulation. (A) D356-2630 B) C700-2058 (C) G842-0420 (D) F403-0203.

The principal component analysis (PCA) was performed to calculate the variance percentage in protein clusters. The dominant movements were observed in the first five eigenvectors in all complexes. The eigenvalues in **D356-2630** complex were 25.6, 41.5, 50.6, 55.6, 66.4 and 78.8% in the first five eigenvectors, respectively. The total variation was 78.8% while the highest fluctuations were observed in the PC1 (25.6%) (Fig 9A). Similarly, the eigen values in the first five eigenvectors of **C700-2058** were 15.5, 28.6, 35.9, 42.1, 57.8, and 75%, respectively. The total variation was 70%. The highest variation was observed in PC1 which recorded 15.5% fluctuations during the simulation (Fig 9B). The eigen values in the first five eigenvectors of **G842-0420** were 23.9,45,52.7,58.7,69.2 and 80.7% and total variation in the complex was 80.7% with the highest fluctuation in PC1 of 23.9% (Fig 9C). Lastly, eigenvalues in **F403-0203** complex 19.1,32.9,39.3,44.8,57.6 and 73% in the first five eigenvectors. The total variation was 73% with the highest fluctuation observed in PC1 19.1% (Fig 9D). The most significant movements are shown with blue regions, with intermediate motions shown by white color, while red color show the minor fluctuations [33]. Further, the PCA based 2D energy surface was also generated to calculate the configurations with stable thermodynamic values. The energy surface calculated the fluctuation direction of energy in two PCs (PC1 and PC2) for carbon alpha atoms. Most of the clusters were found in the local minima well (purple color) which indicated the stable transition of one configuration to another in all four complexes (Fig 10).

#### 3.6.5. MM/GBSA

**Fig 11:**
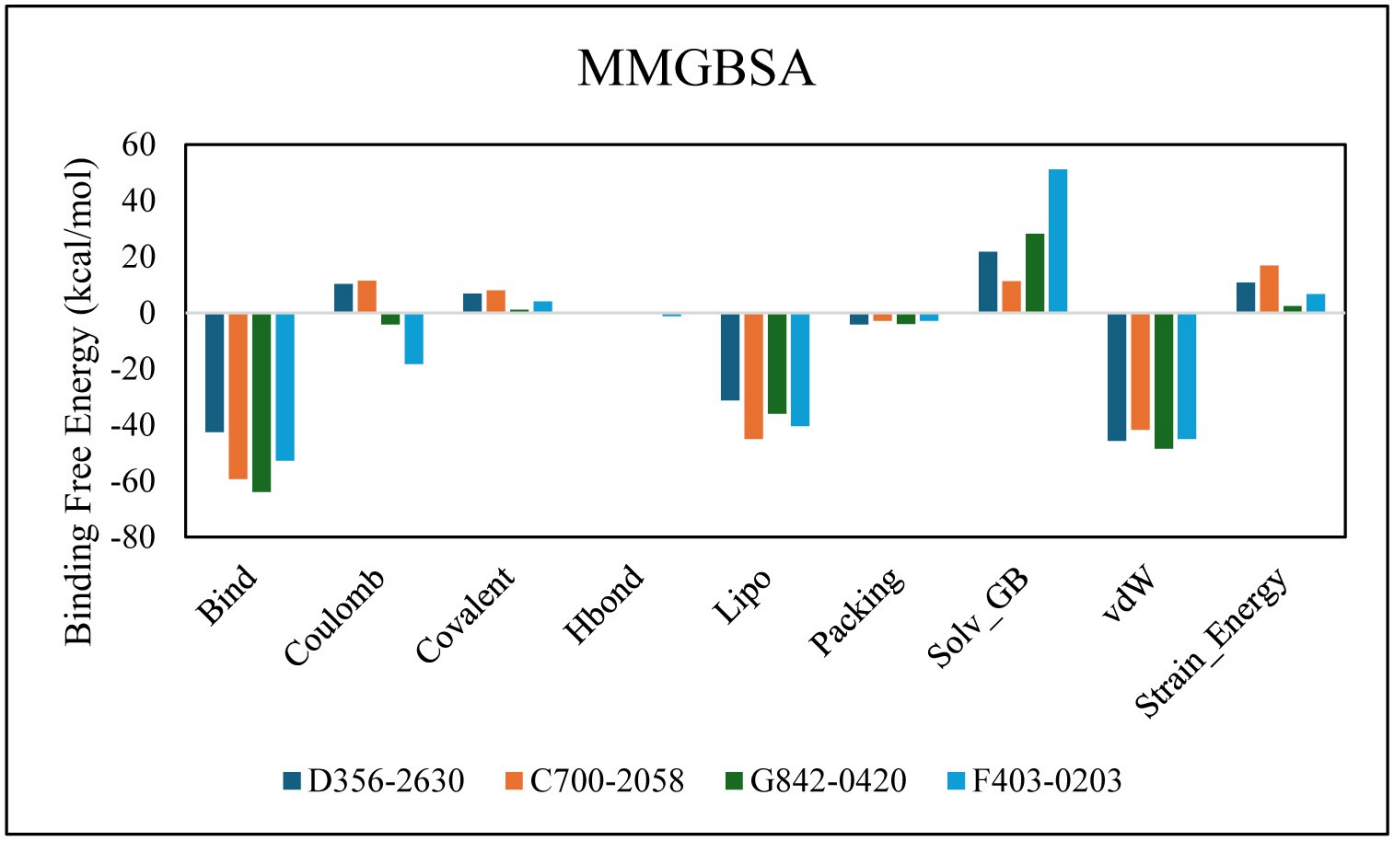
The comparison of binding free energy components in selected complexes.

The prime-MMGBSA module was used to calculate the binding energy of selected complexes [34]. The binding energies of the D356-2630, C700-2058, G842-0420 and F403-0203 complexes were -42.627, -59.33, -63.862, and -52.713kcal/mol, respectively. G_bind_ resulted from non-bonded interactions, G_Coulomb_, G_Packing_, G_Hbond_, G_Lipo_, and G_vdW_ (Fig 11). G_bindLipo_, G_bindvdW_, and G_bindCoulomb_ affected the average binding free energies among all interaction types. Conversely, the final average binding energies were least affected by the G_bindSolvGB_ and G_bindCovalen_t energies. Furthermore, stable hydrogen bonds were observed between the ligands and amino acid residues indicated by G_bindHbond_ interaction values. Thus, the binding energies calculated during simulation supported the binding affinities of ligands obtained during docking studies [35].

## 4. Discussion

Psoriasis is a common chronic immune-mediated disease with numerous comorbidities that impair quality of life. Among psoriasis treatments, phosphodiesterase-4 (PDE4) inhibitors are emerging as new options. PDE4 inhibitors degrade cyclic adenosine monophosphate (cAMP), which contributes to the production of pro-inflammatory mediators [3]. Apremilast, an oral PDE4 inhibitor, is approved for treating psoriasis. While effective, its side effects may limit its utility [36]. Roflumilast, a topical PDE4 inhibitor, was recently approved for psoriasis and has shown promising results in clinical trials [37]. Crisaborole, a PDE4 inhibitor approved for atopic dermatitis, has also been evaluated in psoriasis. These PDE4 inhibitors pave the way for a promising future in psoriasis management [38]. The ongoing expansion of clinical trials, as well as continued research on existing agents and the development of novel inhibitors, have the potential to broaden psoriasis treatment options and improve outcomes [39,40].

In recent years, *in silico* approaches have transformed drug discovery by allowing the identification of novel PDE4 inhibitors with improved efficacy and safety profiles. These *insilico* techniques direct the design and synthesis of novel compounds with enhanced pharmacological properties while enabling the quick and economical exploration of chemical space [41,42]. Several studies have shown that *in silico* approaches can be effective in identifying novel PDE4 inhibitors for psoriasis treatment [43,44]. Hence, this study used molecular docking and virtual screening to identify a group of compounds with a high affinity for PDE4.

The PDE4 crystal structure coordinates (PDB ID: 7W4X) were obtained from the Protein Data Bank. The binding pocket residues were predicted and then chosen to generate the pharmacophore hypothesis, which consisted of seven features (R1981, A456, H1525, D953, D1041, D963, and R2081). By developing a pharmacophore model for PDE4 inhibitors, large chemical libraries can be screened for compounds that share these characteristics. This approach aids in the discovery of structurally diverse compounds that may inhibit PDE4 [45–47].

The CNS library of the ChemDiv database was virtually screened using the created pharmacophore model. A compound was considered a hit during screening if it matched at least four features. Virtual screening is the computational screening of large chemical libraries to find potential drug candidates [48–50]. Forty-six potential hits were identified by using a phase screen cutoff score of 1.5.

The hits generated during virtual screening were prepared and docked to the PDE4 receptor using glide’s SP docking module. The docking poses were assessed using the glide gscore and molecular interactions of the docked compounds. Ten compounds with binding affinities higher than -7 kcal/mol were chosen for further investigation. After analyzing the binding modes of the docked ligands, it was found that each of the ten ligands that were chosen occupied the same area in the protein’s predicted binding pocket [51,52].

The bioactivity scores of the selected ten compounds were predicted. Since bioactivity scores are quantitative indicators of the interaction between putative drug candidates and their biological targets, they are extremely important in the drug discovery process. These rankings aid in the prioritization of compounds for additional research and development according to their anticipated or experimentally verified potency, specificity, and efficacy [53–55]. Certain compounds exhibited near-zero scores, signifying strong binding to the receptors. The results of the analysis showed that the chosen compounds possessed lead compound characteristics.

Further, ADMET characteristics of the selected compounds were analyzed. ADMET is an important set of criteria in the drug discovery and development process. Understanding and optimizing these properties is critical for determining whether a potential drug candidate is both effective and safe for human use [56–59]. Four compounds, D356-2630, C700-2058, G842-0420, and F403-0203, showed better QPPCaco values, so they were selected for further stability analysis.

Selected four compounds were subjected to stability analysis by performing 100ns simulation. These simulations shed light on the dynamics of molecular interactions, structural stability, and time-dependent conformational changes—all essential for comprehending how drugs interact with their intended targets [60–63]. All results show that the protein and ligand complexes remained stable during MD simulation, which offers valuable information about their dynamic behavior and makes them suitable for more research. Binding energies (MMGBSA) of the compounds were also calculated. According to MMGBSA analyses and MD simulations, these compounds were stable as effective inhibitors within the binding pocket of protein. The findings of this study may pave the way for the development of new anti- psoriasis drugs with improved efficacy and safety profiles.

However, computational research has some limitations. These methods rely on the accuracy of the models and algorithms used, which may fail to capture complex biological interactions. Furthermore, the availability and reliability of structural data limit the accuracy of predictions. Computational findings must also be rigorously validated in vivo to ensure efficacy, bioavailability, and safety [64,65]. Despite these limitations, combining CADD approaches with experimental validation shows great promise for developing effective and targeted psoriasis treatments.

## 5. Conclusion

The use of *in silico* techniques in this study successfully identified novel PDE4 inhibitors with promising therapeutic potential for psoriasis. The identified inhibitors had high binding affinities and favorable interactions with the PDE4 enzyme, indicating potential efficacy in modulating inflammatory pathways involved in psoriasis. These findings pave the way for additional experimental validation and optimization, eventually leading to the development of effective treatments for this chronic and debilitating skin condition. The study promotes the continued use of computational tools in drug discovery to address unmet medical needs and improve patient outcomes.
